# Diagnostic accuracy of history taking, physical examination and imaging for phalangeal, metacarpal and carpal fractures: a systematic review update

**DOI:** 10.1186/s12891-019-2988-z

**Published:** 2020-01-07

**Authors:** Patrick Krastman, Nina M. Mathijssen, Sita M. A. Bierma-Zeinstra, Gerald Kraan, Jos Runhaar

**Affiliations:** 1 Department of General Practice, Erasmus MC University Medical Center Rotterdam, Room NA1911 PO Box 2040, 3000 CA Rotterdam, the Netherlands; 2 Department of Orthopaedic Surgery, Reinier de Graaf Groep, Reinier de Graafweg 5-11, 2625 AD Delft, the Netherlands; 3 Department of Orthopaedics, Erasmus MC University Medical Center Rotterdam, Room NA1920 PO Box 2040, 3000 CA Rotterdam, the Netherlands; 4 Department of General Practice, Erasmus MC University Medical Center Rotterdam, Room NA1920 PO Box 2040, 3000 CA Rotterdam, the Netherlands

**Keywords:** Diagnostic tests, Finger, Fracture, Hand, Wrist

## Abstract

**Background:**

The standard diagnostic work-up for hand and wrist fractures consists of history taking, physical examination and imaging if needed, but the supporting evidence for this work-up is limited. The purpose of this study was to systematically examine the diagnostic accuracy of tests for hand and wrist fractures.

**Methods:**

A systematic search for relevant studies was performed. Methodological quality was assessed and sensitivity (Se), specificity (Sp), accuracy, positive predictive value (PPV) and negative predictive value (NPV) were extracted from the eligible studies.

**Results:**

Of the 35 eligible studies, two described the diagnostic accuracy of history taking for hand and wrist fractures. Physical examination with or without radiological examination for diagnosing scaphoid fractures (five studies) showed Se, Sp, accuracy, PPV and NPV ranging from 15 to 100%, 13–98%, 55–73%, 14–73% and 75–100%, respectively. Physical examination with radiological examination for diagnosing other carpal bone fractures (one study) showed a Se of 100%, with the exception of the triquetrum (75%). Physical examination for diagnosing phalangeal and metacarpal fractures (one study) showed Se, Sp, accuracy, PPV and NPV ranging from 26 to 55%, 13–89%, 45–76%, 41–77% and 63–75%, respectively.

Imaging modalities of scaphoid fractures showed predominantly low values for PPV and the highest values for Sp and NPV (24 studies). Magnetic Resonance Imaging (MRI), Computed Tomography (CT), Ultrasonography (US) and Bone Scintigraphy (BS) were comparable in diagnostic accuracy for diagnosing a scaphoid fracture, with an accuracy ranging from 85 to 100%, 79–100%, 49–100% and 86–97%, respectively. Imaging for metacarpal and finger fractures showed Se, Sp, accuracy, PPV and NPV ranging from 73 to 100%, 78–100%, 70–100%, 79–100% and 70–100%, respectively.

**Conclusions:**

Only two studies were found on the diagnostic accuracy of history taking for hand and wrist fractures in the current review. Physical examination was of moderate use for diagnosing a scaphoid fracture and of limited use for diagnosing phalangeal, metacarpal and remaining carpal fractures. MRI, CT and BS were found to be moderately accurate for the definitive diagnosis of clinically suspected carpal fractures.

## Background

Hand and wrist injuries are among the most common traumatic presentations to the emergency department [1, 2], and commonly affect young people of working age [3, 4]. Scaphoid fractures are the most frequently injured carpal bones, accounting for 61–90% of fractures [4–6]. The diagnosis of a scaphoid fracture may however be difficult to establish on a conventional radiograph [7, 8]. Previous research has shown that 10–35% of scaphoid fractures are missed on primary radiographs [4, 9–12]. Metacarpal fractures are detected in 30–40% of all hand fractures in all emergency department admissions [4, 9, 10].

Hand and wrist injuries represent a considerable economic burden, with high health-care and productivity costs [13]. The total costs have been estimated at US $410 million per year, with US $307 million in productivity costs [14].

If not treated properly, patients with hand and wrist injuries may experience lifelong pain and lose their job, which also has major effects on their quality of life [15]. Accurate diagnosis and early treatment of hand and wrist fractures are important because missed diagnosis and delayed initiation of therapy increase the risk of complications and subsequent functional impairment [16–22].

In recent decades, research has predominantly focused on imaging modalities for the diagnosis of wrist fractures. However, the standard diagnostic work-up for wrist complaints that are suspected fractures should also include detailed patient history taking, a conscientious physical examination and, only if needed, imaging [23]. It has been shown that different provocative tests are somewhat useful for diagnosing wrist fractures [24–27], but there is no consensus on imaging protocols due to limited evidence regarding the diagnostic performance of these advanced imaging techniques [28]. Therefore, diagnosing wrist pathologies remain complex and challenging and there is increasing demand for evidence for accurate diagnostic tools [29].

Diagnostic studies performed in hospital care cannot automatically be translated into guidelines for non-institutionalized general practitioner care [30]. The clinical utility of diagnostic tests for hand and wrist fractures is hindered by the low prevalence of true fractures, approximately 7% on average [31].

Currently, there are several systematic reviews available on the diagnostic accuracy of tests for the diagnosis of hand and wrist fractures, as presented in Table 1 [32–39]. Of these, only the review by Carpenter et al. used ‘history’ as a keyword in their search terms, but they could not find studies assessing the diagnostic accuracy of history for scaphoid fractures [32]. All the available systematic reviews only examined diagnostic tests for scaphoid fractures [32–39], while in practice it is often not quite clear during the diagnostic process which hand or wrist anatomical structure or tissue (soft tissue or bone) is affected. Moreover, these reviews focused predominantly on imaging as a diagnostic tool, while in clinical practice a diagnosis is mainly made on history taking and physical examination.

**Table 1:** Characteristics of the Currently Available Systematic Reviews on the Diagnostic Accuracy of Tests

Author(s)	Population in eligible studies as described by the review authors	Fracture	Number of studies included	Diagnostic test	Pooled Se (95% CI)	Pooled Sp (95% CI)	Positive LR	Conclusion
HISTORY TAKING								
Carpenter (2014) [32]	Emergency Department.	Scaphoid	0					History examination alone is inadequate to rule in or rule out scaphoid fracture.
PHYSICAL EXAMINATION								
Carpenter (2014) [32]	Emergency Department.	Scaphoid	6	ASB tenderness	0.96 (0.92–0.98)	0.39 (0.36–0.43)		Except for the absence of snuffbox tenderness, which can significantly reduce the probability of scaphoid fracture, physical examination alone is inadequate to rule in or rule out scaphoid fracture.
			6	LTC	0.82 (0.77–0.87)	0.58 (0.54–0.62)		
			7	Ultrasound fibration pain	0.67 (0.59–0.75)	0.57 (0.51–0.62)		
			3	Clamp sign	0.73 (0.67–0.78)	0.92 (0.89–0.95)		
			3	Painfull ulnar deviation	0.77 (0.68–0.83)	0.42 (0.34–0.49)		
			3	STT	0.92 (0.86–0.96)	0.47 (0.43–0.52)		
			2	Resisted supination pain	0.94 (0.85–0.98)	0.74 (0.63–0.84)		
Burrows (2014) [33]	Not specified	Scaphoid	5	ASB tenderness			1.52 (1.12–2.06)	Three clinical tests with statistically significant diagnostic validity were identified. In isolation, the clinical significance of each is questionable.
			7	Scaphoid compression test			2.37 (1.27–4.41)	
			3	STT			1.67 (1.33–2.09)	
Mallee (2015) [34]	Patients presenting to the emergency department or outpatient clinic	Scaphoid	8	ASB tenderness	0.87–1.00 ^a^	0.03–0.98 ^b^		Anatomical snuff box tenderness was the most sensitive clinical test. The low specificity of the clinical tests may result in a considerable number of over-treated patients. Combining tests improved the post-test fracture probability.
			8	LTC	0.48–1.00 ^a^	0.22–0.97 ^b^		
			4	STT	0.82–1.00 ^a^	0.17–0.57 ^b^		
			4	Painfull ulnar deviation	0.67–1.00 ^a^	0.17–0.60 ^b^		
			4	ASB swelling	0.67–0.77 ^a^	0.37–0.72 ^b^		
IMAGING								
Carpenter (2014) [32]	Emergency Department.	Scaphoid	5	X-ray fat pad	0.82 (0.76–0.86)	0.72 (0.68–0.75)		MRI is the most accurate imaging test to diagnose scaphoid fractures in ED patients with no evidence of fracture on initial x-rays. If MRI is unavailable, CT is adequate to rule in scaphoid fractures, but inadequate for ruling out scaphoid fractures.
			18	BS	0.91 (0.87–0.94)	0.86 (0.83–0.88)		
			6	US	0.80 (0.67–0.90)	0.87 (0.81–0.91)		
			8	CT	0.83 (0.83–0.89)	0.97 (0.94–0.98)		
			13	MRI	0.96 (0.92–0.99)	0.98 (0.96–0.99)		
Yin (2012) [35]	Not specified	Scaphoid	28	Follow-up radiographs	0.91 (0.81–0.98)	1.00 (0.99–1.00)		If we acknowledge the lack of a reference standard for diagnosing suspected scaphoid fractures, MRI is the most accurate test; follow-up radiographs and CT may be less sensitive, and bone scintigraphy less specific.
			18	BS	0.98 (0.96–0.99)	0.94 (0.91–0.95)		
			15	MRI	0.98 (0.95–0.99)	1.00 (0.99–1.00)		
			9	CT	0.85 (0.74–0.94)	1.00 (0.98–1.00)		
Yin (2010) [36]	Not specified	Scaphoid	15	BS	0.97 (0.93–0.99)	0.89 (0.83–0.94)		Bone scintigraphy and MRI have equally high sensitivity and high diagnostic value for excluding scaphoid fracture; however, MRI is more specific and better for confirming scaphoid fracture.
			10	MRI	0.96 (0.91–0.99)	0.99 (0.96–1.00)		
			6	CT	0.93 (0.83–0.98)	0.99 (0.96–1.00)		
Mallee (2014) [34]	People of all ages who presented at hospital or clinic	Scaphoid	6	BS	0.99 (0.69–1.00)	0.86 (0.73–0.94)		Bone scintigraphy is statistically the best diagnostic modality to establish a definitive diagnosis in clinically suspected fractures when radiographs appear normal. The number of overtreated patients is substantially lower with CT and MRI.
			4	CT	0.72 (0.36–0.92)	0.99 (0.71–1.00)		
			5	MRI	0.88 (0.64–0.97)	1.00 (0.38–1.00)		
Kwee (2018) [37]	Not specified	Scaphoid	7	US	0.86 (0.74–0.93)	0.84 (0.72–0.91)		Ultrasound can diagnose radiographically occult scaphoid fracture with a fairly high degree of accuracy.
Ali (2018) [38]	Not specified	Scaphoid	6	US	0.94 (0.78–1.00)	0.89 (0.78–1.00)		US reveals high sensitivity and specificity in scaphoid fracture diagnosis.

*ASB* Anatomic snuff-box, *LTC* Longitudinal (thumb) compression test, *STT* Scaphoid tubercle tenderness, *BS* Bone Scintigraphy, *US* Ultrasound, *CT* Computed TomographyMRI: Magnetic Resonance Imaging

^a^Sensitivity range described, because of the high heterogeneity Mallee et al. [34] refrained from calculating pooled estimate points

^b^Specificity Range described, because of the high heterogeneity Mallee et al. [34] refrained from calculating pooled estimate points

Therefore, the purpose of this literature review is to provide an up-to-date systematic overview of the diagnostic accuracy of history taking, physical examination and imaging for phalangeal, metacarpal and carpal fractures and to distinguishing between studies in hospital and non-institutionalized general practitioner care settings, as test properties may differ between settings. Compared to previously published reviews, in this systematic review we also included studies that examined history taking and physical examination for phalangeal, metacarpal or carpal fractures.

## Methods

### Data sources and searches

A review protocol was drafted, but central registration was not completed. The Preferred Reporting Items for Systematic Reviews and Meta-Analyses (PRISMA) Statement was used to guide the conduct and reporting of the study [40]. A Biomedical Information specialist (Wichor M. Bramer) performed a search for studies in Medline, Embase, Cochrane Library, Web of Science, Google Scholar ProQuest and Cinahl from 2000 up to 6 February 2019. This starting point was used since multiple reviews are available that already cover the period up to the year 2000 (Table 1). Search terms included phalangeal, metacarpal and carpal injuries, anamnestic assessment, provocative test(s), diagnostic test(s) and imaging tests. The full electronic search strategy for the Embase database is presented in Table 2 (the others are available upon request).

**Table 2:** Example electronic search strategy

Database	Search terms
Embase	(‘hand injury’/exp. OR ‘wrist injury’/exp. OR ‘wrist fracture’/exp. OR ((‘hand bone’/exp. OR wrist/exp. OR hand/exp. OR ‘wrist pain’/exp. OR ‘hand pain’/exp) AND (‘bone injury’/exp. OR fracture/de OR ‘ligament injury’/exp. OR ‘ligament rupture’/exp)) OR (((hand OR hands OR wrist* OR finger* OR carpal* OR carpus OR phalanx* OR metacarp* OR capitate* OR hamat* OR lunat* OR pisiform* OR scaphoid* OR trapezium* OR trapezoid* OR triquetr* OR navicular* OR lunar OR semilunar* OR multangulum* OR pyramid* OR metacarpophalang* OR thumb* OR ‘distal radius’ OR ‘distal ulna’ OR ‘distal radial’ OR ‘distal ulnar’ OR scapholunate* OR lunotriquetral* OR ‘triangular fibrocartilaginous’ OR SLIL OR LTIL OR tfcc OR ‘ulnar collateral ligament’ OR ‘ulnar collateral ligaments’ OR ucl) NEAR/3 (injur* OR trauma* OR wound* OR lesion* OR dislocate* OR fracture* OR damage* OR tear* OR sprain* OR displace* OR rupture*))):ab,ti) AND (‘diagnostic test’/de OR ‘function test’/exp. OR ‘diagnostic error’/exp. OR ‘diagnostic accuracy’/exp. OR ‘diagnostic value’/exp. OR ‘differential diagnosis’/exp. OR ‘delayed diagnosis’/exp. OR ‘sensitivity and specificity’/exp. OR (((diagnos* OR detect* OR differen* OR strength* OR motion*) NEAR/3 (test* OR accura* OR error* OR false OR fail* OR value* OR impact* OR effective* OR earl* OR missed OR correct* OR incorrect* OR delay* OR difficult* OR negative* OR positive* OR sensitivit* OR specificit* OR confirm* OR abilit*)) OR (diagnos* NEAR/3 differen*) OR misdiagnos* OR underdiagnos* OR undetect* OR (predict* NEAR/3 value*) OR (function* NEAR/3 test*) OR (false NEAR/3 (negative* OR positive*))):ab,ti) NOT ([Conference Abstract]/lim OR [Letter]/lim OR [Note]/lim OR [Editorial]/lim) AND [english]/lim NOT ([animals]/lim NOT [humans]/lim)

Search terms for the other databases are available upon request

### Study selection

Studies describing diagnostic accuracy of history taking, physical examination or imaging in adult patients (age ≥ 16 years) with phalangeal, metacarpal and/or carpal fractures were included. No language restriction was applied. Case reports, reviews and conference proceedings were excluded. Distal radius and ulna injuries were also excluded, as they can be diagnosed accurately with plane X-ray or computer tomography imaging.

Two reviewers (PK, YA) read all titles and abstracts independently. Articles that could not be excluded on the basis of the title and/or abstract were retrieved in full text and were read and checked for inclusion by the two reviewers independently. If there was no agreement, a third reviewer (JR) made the final decision. In addition, the reference lists of all included studies were reviewed to check for additional relevant studies.

### Data extraction and methodological quality assessment

Two reviewers (PK, JR) independently extracted the data. Data were extracted describing the study design, characteristics of the study population, test characteristics, study population setting (hospital care or non-institutionalized general practitioner care) and diagnostic parameters. Methodological quality was assessed by two independent reviewers (PK, JR), using the Quality Assessment of Diagnostic Accuracy Studies (QUADAS-2) checklist [41]. Disagreements were resolved by discussion.

### Heterogeneity

Key factors in a meta-analysis are the number and the methodological quality of the included studies and the degree of heterogeneity in their estimates of diagnostic accuracy [42]. Heterogeneity in diagnostic test accuracy reviews is expected and the possibilities of performing meta-regression analyses will depend on the number of studies available for a specific index test that provide sufficient information [39]. The data from the included studies were combined when studies showed no limitations according to QUADAS-2 and had no other forms of bias (e.g. incorporation bias).

### Data synthesis and analysis

The following values were extracted, if documented: sensitivity (Se), specificity (Sp), accuracy, positive predictive value (PPV), negative predictive value (NPV) and likelihood ratio (LR). If these diagnostic outcomes were not reported, they were calculated using published data. If an included study presented results from multiple independent observers, the measures of Se, Sp, accuracy, PPV and NPV were averaged over the observers.

### Index test

Diagnostic tools such as history taking, physical examination or imaging were accepted as index tests.

### Reference standard

There is no consensus about the reference test for the diagnosis of a true fracture of the phalangeal, metacarpal or carpal bones [35]. Therefore, in this systematic review clinical outcome (physical examination or additional treatment) and/or various (combined) imaging modalities during follow-up were used as the reference standard for confirming diagnosis of phalangeal, metacarpal or carpal fractures.

## Results

**Fig. 1:**
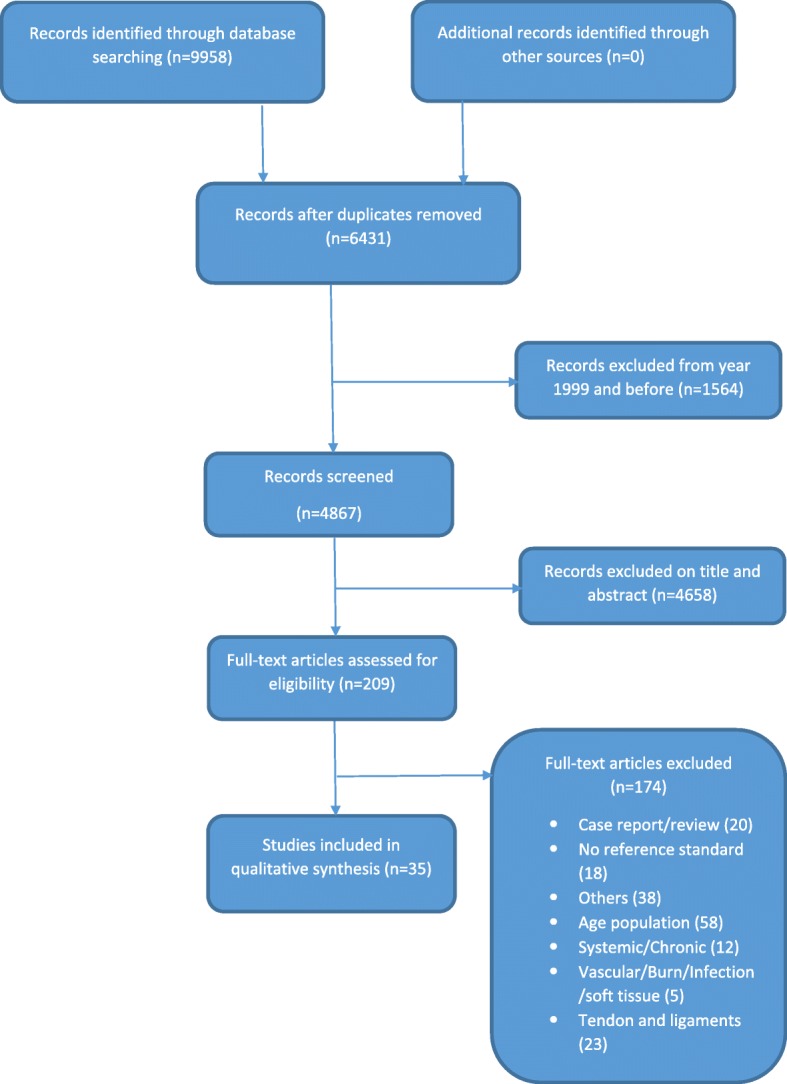
Flow chart study selection

The flow diagram is presented in Fig. 1. A total of 35 diagnostic studies were identified, assessed and interpreted. The characteristics of these studies are presented in Table 3. 20 studies were performed in an emergency department, four studies in a traumatology setting and three other studies in a radiology department. The patients in the studies by Mallee et al. [56–58] were derived from one prospective study; therefore the setting was the same for each study: patients were initially seen by the emergency physicians and in follow-up by the orthopaedic department and/or trauma surgery department, depending on who was on call. In five studies the setting was not specified. To our knowledge, all first authors of those five studies were working in a hospital care setting, so we assume all to have been done in hospital care. History taking, physical examination and imaging as index tests were investigated in 0, 20% (7/35) [48, 53, 62, 64, 67, 73, 77] and 86% (30/35) [43–47, 49–51, 53–61, 63, 65, 66, 68–77] of the studies, respectively.

**Table 3:** Characteristics of the Eligible Studies (*N* = 35)

Author(s)	Participants	Design	Department of patient presentation (Country)	Fracture	Index test	Reference test
SCAPHOID AND OTHER CARPAL BONES FRACTURES						
Adey (2007) [43]	30	Retrospective	Not described (USA)	Scaphoid	CT	Radiographs 6 weeks after injury
Annamalai (2003) [44]	50	Retrospective	Not described (Scotland)	Scaphoid	Radiology (scaphoid and pronator fat stripe)	MRI 0,2 T (12-72 h)
Behzadi (2015) [45]	124	Retrospective	Emergency department (Germany)	Scaphoid	Radiographs (anterior-posterior, lateral and oblique projections)	MDCT (within 10 days)
Beeres (2007) [46]	50	Prospective	Emergency department (Netherlands)	Scaphoid and other carpal bones	Bone scintigraphy (3–7 days after injury)	Clinical outcome: physical examination at fixed intervals No fracture, with a normal physical examination at 2 or 6 weeks, BS was considered correct. However, if there were clinical signs of a fracture after 2 and 6 weeks, BS was considered false negative. Another fracture in the carpal region and physical examination after 2 weeks (during change of cast) matched with such a fracture, BS was considered correct. But, when physical examination after 2 weeks showed no signs of fracture, BS was considered false positive. A scaphoid fracture, confirmed on physical examination after 2 weeks (during change of cast), BS was considered correct. If however, neither physical examination after 2 weeks, nor consecutive physical examinations showed evidence of a scaphoid fracture, there was no scaphoid fracture. BS was then considered false positive.
Beeres (2008) [47]	100	Prospective	Emergency department (Netherlands)	Scaphoid	MRI 1.5 T (< 24 h) and Bone scintigraphy (between 3 and 5 days)	Absence or presence of a fracture on both MRI and bone scintigraphy, or in the case of discrepancy, clinical and/or radiological evidence of a fracture.
Bergh (2014) [48]	154	Prospective	Emergency department, outpatient clinic (Norway)	Scaphoid	Clinical Scaphoid Score (CSS): tenderness in the anatomical snuffbox with the wrist in ulnar deviation (3 points) + tenderness over the scaphoid tubercle (2 points) + pain upon longitudinal compression of the thumb (1 point)	MRI 1.5 T
Breederveld (2004) [49]	29	Prospective	Emergency department (Netherlands)	Scaphoid	BS (three-fase) and CT	Clinical follow-up (including CT and Bone scintigraphy)
Cruickshank (2007) [50]	47	Prospective	Teaching emergency department (Australia)	Scaphoid and other carpal bones	CT (same or next day)	The diagnosis on Day 10 with clinical examination and X-rays, with MRI performed in patients with persistent tenderness but normal X-rays.
Fusetti (2005) [51]	24	Prospective	Not described (Switzerland)	Scaphoid	HSR-S (< 24 h of the clinical examination)	CT (immediately after HSR-S performed)
Gabler (2001) [52]	121	Prospective	Department of traumatology: fracture clinics (Austria)	Scaphoid	Repeated clinical examination (tenderness over the anatomical snuff box or the carpus as well as a positive scaphoid compression test) and radiological examinations (scaphoid views)	MRI 1.0 T
Herneth (2001) [53]	15	Prospective	Not described (Austria)	Scaphoid	Clinical examination, radiography and High-spatial resolution ultrasonography	MRI 1,0 T (< 72 h)
Ilica (2011) [54]	54	Prospective	Emergency department (Turkey)	Scaphoid	MDCT	MRI 1.5 T
Kumar (2005) [55]	22	Prospective	Collaboration between the Department of Emergency Medicine and Medical Imaging (New Zealand)	Scaphoid	MRI 1.5 T (< 24 h)	MRI in those without fracture at MRI < 24 h or no clinical signs of fracture
Mallee (2011) [56]	34	Prospective	Initially emergency physicians and in follow-up by the Orthopedic department and/or Trauma surgery department, depending on who was on call. (Netherlands)	Scaphoid	CT and MRI 1.0 T (within 10 days)	Radiographs, after 6 weeks follow-up
Mallee (2016) [57]	34	Prospective	Initially emergency physicians and in follow-up by the Orthopedic department and/or Trauma surgery department, depending on who was on call. (Netherlands)	Scaphoid	6-weeks radiographs in JPEG- and DICOM- view	CT, MRI, or CT and MRI
Mallee (2014) [58]	34	Prospective	Initially emergency physicians and in follow-up by the Orthopedic department and/or Trauma surgery department, depending on who was on call. (Netherlands)	Scaphoid	CT-scaphoid: reformations in planes defined by the long axis of the scaphoid. CT-wrist: reformations made in the anatomic planes of the wrist. CT performed within 10 days.	Radiographs in four standard scaphoid views after 6 weeks follow-up.
Memarsadeghi (2006) [59]	29	Prospective	Not described (Austria)	Scaphoid	MDCT and MRI 1,0 T	Radiographs obtained 6 weeks after trauma. View: posteroanterior with the wrist in neutral position, lateral, semipronated oblique scaphoid, and radial oblique scaphoid.
Ottenin (2012) [60]	100	Retrospective	Radiology department of the emergency unit (France)	Scaphoid and other carpal bones	Tomosynthesis (frontal and lateral), MDCT (within 7 days) and radiographs (posteroanterior view, lateral view, anteroposterior oblique view, scaphoid view with ulnar deviation, and posteroanterior view with clenched fist)	The reference standard for each case was determined after completion of all examinations; analysis of MRI (*n* = 13; performed in cases of doubt after completion of diagnostic standard radiography, tomosynthesis, and CT); and follow-up information obtained by physical examination or, in case of no clinical follow-up, by telephone recalls.
Platon (2011) [61]	62	Prospective	Emergency department (Switzerland)	Scaphoid	US within 3 days (presence of a cortical interruption of the scaphoid along with a radio-carpal or scaphotrapezium-trapezoid effusion)	CT (immediately after US)
Rhemrev (2010) [62]	100	Prospective	Emergency department (Netherlands)	Scaphoid	MDCT (< 24 h) and Bone scintigraphy (3–5 days)	Final diagnosis after final discharge, according to the following standard: If CT and bone scintigraphy showed a fracture, the final diagnosis was fracture. If CT and bone scintigraphy showed no fracture, the final diagnosis was no fracture. In case of discrepancy between CT and bone scintigraphy, both radiographic (6 weeks after injury) and physical reevaluation during follow-up were used to make a final diagnosis. In case of radiographic evidence of a scaphoid fracture 6 weeks after injury, the final diagnosis was fracture. In case of no radiographic evidence of a scaphoid fracture 6 weeks after injury but there were persistent clinical signs of a scaphoid fracture after 2 weeks, the final diagnosis was fracture. If there was no radiographic evidence of a scaphoid fracture 6 weeks after injury and there were no longer clinical signs of a scaphoid fractures throughout follow-up, the final diagnosis was no fracture.
Rhemrev (2010) [63]	78	Prospective	Emergency department (Netherlands)	Scaphoid	Three clinical exams: 1) inspection of the snuffbox for the presence of ecchymosis or edema, 2) flexion and extension of the wrist, 3) Supination and pronation strength, 4) Grip strength.	MRI 1,5 T, bone scintigraphy, radiography and physical re-evaluation during 6 weeks clinical follow-up.
Steenvoorde (2006) [64]	31	Not described	Emergency department (Netherlands): request for radiograph of the scaphoid by general practitioners were excluded	Scaphoid and other carpal bones	Five or more positive clinical tests out of seven tests: 1) loss of concavity of the anatomic snuff box, 2) snuffbox tenderness, 3) the clamp sign, 4) palmar tenderness of the scaphoid, 5) axial compression of the thumb along its longitudinal axis, 6) site of pain on resisted supination, 7) site of pain on ulnar deviation.	Clinical follow-up
Yildirim (2013) [65]	63	Prospective	Emergency department (Turkey)	Scaphoid	BUS (presence of a cortical interruption of the scaphoid along with a radiocarpal or scaphotrapezium trapezoid effusion)	MRI (< 24 h)
de Zwart (2016) [66]	33	Prospective	Emergency department (Netherlands)	Scaphoid	MRI (< 72 h), CT(< 72 h) and Bone Scintigraphy (between 3 and 5 days)	If MRI, CT and BS all showed a fracture, the final diagnosis was: fracture. If MRI, CT and BS all showed no fracture, the final diagnosis was: no fracture. In case of discrepancy between MRI, CT and BS, the final diagnosis was established based on specific clinical signs of a fracture after 6 weeks (tender anatomic snuffbox and pain in the snuffbox when applying axial pressure on the first or second digit) combined with the radiographic evidence of a fracture after 6 weeks. If these signs were absent and no radiographic evidence, the final diagnosis was: no fracture.
Sharifi (2015) [67]	175	Prospective	Emergency department (Iran)	Scaphoid fractures	VAS pain score (anatomical snuff box tenderness)	MRI
Brink (2014) [68]	98	Prospective	Department of Radiology (Netherlands)	Fractures carpus and metacarpal	CT or radiography	Clinical follow-up
Neubauer (2018) [69]	102	Retrospective	Orthopedics and Trauma/Hand Surgery (Germany)	Scaphoid fractures	CBCT or radiography	Clinical follow-up (including images)
Borel (2017) [70]	49	Prospective	Orthopedics and Trauma Surgery (France)	Scaphoid or wrist fractures	CBCT	MRI
SCAPHOID, OTHER CARPAL AND METACARPAL BONES FRACTURES						
Balci (2015) [71]	455	Retrospective	Emergency department (Turkey)	Carpal and metacarpal	Radiographs	MDCT
Jorgsholm (2013) [72]	296	Prospective	Emergency department (Sweden)	Scaphoid, other carpal and metacarpal bones	Radiographs (dorsovolar and lateral projections with an additional 4 views of the scaphoid.) and CT	MRI 0.23 T (within 3 days)
Nikken (2005) [73]	87	Prospective	Radiology department referred by traumatologist, orthopedic surgeon or emergency physician (Netherlands)	Scaphoid and other carpal bones. Metacarpal bones II–IV	Anatomic snuffbox tenderness, radiographs (posteroanterior and lateral projection) and MRI 0,2 T (short procedure)	Additional treatment
CARPAL AND METACARPAL BONES AND PHALANGEAL FRACTURES						
Javadzadeh (2014) [74]	260	Not described	Emergency department (Iran)	Carpal, metacarpal, and phalangeal	BUS and WBT ultrasonography	Radiographs (not described when performed)
METACARPAL BONES AND/OR PHALANGEAL FRACTURES						
Faccioli (2010) [75]	57	Prospective	Traumatology department (Italy)	Phalangeal	CBCT	MSCT
Kocaoglu (2016) [76]	96	Prospective	Emergency department (Turkey)	Metacarpal	US	Radiographs (anteroposterior and oblique)
Tayal (2007) [77]	78	Prospective	Emergency department (USA)	Metacarpal and phalangeal	US and physical examination	Radiographs and when operated, surgical findings

*MRI* Magnetic resonance imaging, *CT* Computed Tomography, *CBCT* Cone Beam Computed Tomography, *MSCT* Multi-slice Computed Tomography, *HSR-S* High Spatial Resolution sonography, *BUS* Bedside ultrasonography, *WBT* Water bath technique *ROM* Range of motion

### Quality assessment

There was considerable underreporting of important quality domains in 23 of the 35 studies (see Table 4). In 13 of the 35 studies [43, 44, 48, 50, 54, 55, 59, 64, 67, 72, 74, 76, 77], patient selection was not well documented. Furthermore, the risk of bias was predominantly due to the absence of a proper description of the index test (9/35) [43, 45, 49, 53, 55, 64, 65, 72, 77] or the reference standard (13/35) [45, 49, 55, 62, 64–68, 71–73, 75]. Twelve of the studies (34%) demonstrated no limitations when risk of bias was assessed, according to QUADAS-2 [46, 47, 51, 52, 56–58, 60, 61, 63, 69, 70]. Eight showed incorporation bias [46, 47, 49, 55, 60, 62, 66, 69].

**Table 4:** Summary of Methodological Quality according to Quality Assessment of Diagnostic Accuracy Studies-2

Author(s)	Risk of Bias				Applicability Concerns		
	Patient Selection	Index Test	Reference standard	Flow and Timing	Patient Selection	Index Test	Reference standard
Adey (2007) [43]	**HR**	***UR***	LR	LR	LR	LR	LR
Annamalai (2003) [44]	**HR**	LR	LR	LR	LR	LR	LR
Balci (2015) [71]	LR	LR	**HR**	LR	LR	LR	LR
Beeres (2007) [46]	LR	LR	LR	LR	LR	LR	LR
Beeres (2008) [47]	LR	LR	LR	LR	LR	LR	LR
Behzadi (2015) [45]	LR	**HR**	**HR**	LR	LR	LR	LR
Bergh (2014) [48]	***UR***	LR	LR	LR	LR	LR	LR
Borel (2017) [70]	LR	LR	LR	LR	LR	LR	LR
Breederveld (2004) [49]	LR	***UR***	***UR***	LR	LR	LR	LR
Brink (2019) [68]	LR	LR	**HR**	LR	LR	LR	LR
Cruickshank (2007) [50]	***UR***	LR	LR	LR	LR	LR	LR
Faccioli (2010) [75]	LR	**HR**	**HR**	LR	LR	LR	LR
Fusetti (2005) [51]	LR	LR	LR	LR	LR	LR	LR
Gabler (2001) [52]	LR	LR	LR	LR	LR	LR	LR
Herneth (2001) [53]	LR	***UR***	LR	LR	LR	LR	LR
Ilica (2011) [54]	***UR***	LR	LR	LR	LR	LR	LR
Javadzadeh (2014) [74]	***UR***	LR	LR	LR	LR	LR	LR
Jorgsholm (2013) [72]	***UR***	**HR**	**HR**	LR	LR	LR	LR
Kocaoglu (2016) [76]	***UR***	LR	LR	LR	LR	LR	LR
Kumar (2005) [55]	***UR***	**HR**	**HR**	**HR**	LR	LR	LR
Mallee (2011) [56]	LR	LR	LR	LR	LR	LR	LR
Mallee (2016) [57]	LR	LR	LR	LR	LR	LR	LR
Mallee (2014) [58]	LR	LR	LR	LR	LR	LR	LR
Memarsadeghi (2006) [59]	***UR***	LR	LR	LR	LR	LR	LR
Neubauer (2018) [69]	LR	LR	LR	LR	LR	LR	LR
Nikken (2005) [73]	LR	LR	**HR**	LR	LR	LR	LR
Ottenin (2012) [60]	LR	LR	LR	LR	LR	LR	LR
Platon (2011) [61]	LR	LR	LR	LR	LR	LR	LR
Rhemrev (2010) [62]	LR	LR	**HR**	LR	LR	LR	LR
Rhemrev (2010) [63]	LR	LR	LR	LR	LR	LR	LR
Sharifi (2015) [67]	***UR***	LR	***UR***	LR	LR	LR	LR
Steenvoorde (2006) [64]	***UR***	**HR**	**HR**	LR	LR	LR	LR
Tayal (2007) [77]	***UR***	LR	LR	LR	LR	LR	LR
Yildirim (2013) [65]	LR	**HR**	**HR**	**HR**	LR	LR	LR
de Zwart (2016) [66]	LR	LR	**HR**	LR	LR	LR	LR

Abbreviations: *LR* Low Risk, *HR* High Risk, *UR* Unclear Risk

### Diagnosing carpal fractures in hospital care

Table 5 presents the accuracy of the diagnostic tests of all the carpal fractures. Two studies described the diagnostic accuracy of history taking [62, 67]. Physical examination [48, 53, 62, 64] and combined physical and radiological examination [52] for diagnosing scaphoid fractures showed Se, Sp, accuracy, PPV and NPV ranging from 15 to 100%, 13–98%, 55–73%, 14–73% and 75–100%, respectively.

**Table 5:** Diagnostic Accuracy of the Diagnostic Tests of the Carpal, Metacarpal and Phalangeal Fractures (*N*=35)

Author(s)	Index test	Reference test	Fracture	Se % (95% CI)	Sp % (95% CI)	Accuracy % (95% CI)	PPV % (95% CI)	NPV % (95% CI)
Scaphoid and other carpal bones fractures								
History taking								
Sharifi (2015) [74]	VAS pain score cutt of: 3,0	MRI	Scaphoid	100	100			
	4,5	MRI	Scaphoid	94	92			
	5,5	MRI	Scaphoid	94	82			
	6,5	MRI	Scaphoid	94	72			
	7,5	MRI	Scaphoid	88	43			
	8,5	MRI	Scaphoid	75	28			
	9,5	MRI	Scaphoid	31	13			
Physical examination								
Bergh (2014) [44]	Clinical Scaphoid Score ≥4	MRI 1,5T	Scaphoid	77	56	58	14	96
Gabler (2001) [45]	Repeated clinical and radiological examinations (after 10 days)	MRI 1,0T	Scaphoid	82				
	Repeated clinical and radiological examinations (after 38 days)	MRI 1,0T	Scaphoid	100	100	100	100	100
	Repeated clinical and radiological examinations (after 38 days)	MRI 1,0T	Capitate	100				
	Repeated clinical and radiological examinations (after 38 days)	MRI 1,0T	Triquetrum	75				
	Repeated clinical and radiological examinations (after 38 days)	MRI 1,0T	Hamate	100				
	Repeated clinical and radiological examinations (after 38 days)	MRI 1,0T	Lunate	100				
	Repeated clinical and radiological examinations (after 38 days)	MRI 1,0T	Trapezoid	100				
Herneth (2001) [47]	Clinical examination	MRI	Scaphoid	89	50	73	73	75
Rhemrev (2010) [63]	Pronation strength ≤10%	Clinical follow-up	Scaphoid	69	65			
	Extension < 50%	Clinical follow-up	Scaphoid	85	59			
	Supination strength ≤10%	Clinical follow-up	Scaphoid	85	77			
	Grip strength ≤25%	Clinical follow-up	Scaphoid	92	34			
	extension <50%, supination strength <10% and presence of a previous fracture of either the involved or uninvolved hand or wrist.	Clinical follow-up	Scaphoid	15	98		61	85
	extension <50%, supination strength <10% and presence of a previous fracture of either the involved or uninvolved hand or wrist.	Clinical follow-up	No scaphoid fracture	46	92		54	89
Steenvoorde (2006) [64]	Seven clinical tests (≥ 5 positive tests)	Clinical follow-up	Scaphoid	100	13	55	52	100
Imaging: Radiographs								
Annamalai (2003) [44]	Scaphoid fat stripe on radiography	MRI 0,2T (12-72h)	Scaphoid	50	50	50	50	50
	Pronator fat stripe on radiography		Scaphoid	26	70	48	46	49
Balci (2015) [71]	Radiographs	MDCT	Scaphoid	66	98		77	96
	Radiographs	MDCT	Lunate	20	100		100	97
	Radiographs	MDCT	Triquetrum	29	100		100	96
	Radiographs	MDCT	Pisiform	0	100		0	99
	Radiographs	MDCT	Trapezium	18	99		33	98
	Radiographs	MDCT	Trapezoid	0	100		0	99
	Radiographs	MDCT	Capitate	8	100		50	98
	Radiographs	MDCT	Hamata	41	100		78	98
Behzadi (2015) [45]	Radiographs (anterior-posterior, lateral and oblique projections)	MDCT (within 10 days)	Scaphoid	43	81	60	53	73
Herneth (2001) [53]	Radiographs	MRI	Scaphoid	56	100	73	100	60
Jorgsholm (2013) [72]	Radiographs	MRI 0.23T (within 3 days)	Scaphoid	70 (61-78)	98 (95-100)	87	97	82
	Radiographs 6-week: DICOM viewer	MRI 0.23T (within 3 days)	Triquetrum	59 (33-82)				
	Radiographs 6-week: DICOM viewer	MRI 0.23T (within 3 days)	Lunate	25 (1-81)				
	Radiographs 6-week: DICOM viewer	MRI 0.23T (within 3 days)	Capitate	7 (0-34)				
	Radiographs 6-week: DICOM viewer	MRI 0.23T (within 3 days)	Hamata	0 (0-46)				
Mallee (2016) [57]	Radiographs 6-week: JPEG	MRI	Scaphoid	42 (37-47)	56 (54-59)	53 (51-56)	20 (17-23)	79 (76-81)
	Radiographs 6-week: JPEG	MRI	Scaphoid	64 (57-71)	53 (50-57)	56 (52-59)	26 (22-30)	85 (82-88)
Mallee (2016) [57]	Radiographs 6-week: JPEG	CT	Scaphoid	56 (50-62)	59 (56-61)	58 (56-61)	19 (16-22)	89 (87-90)
Mallee (2016) [57]	Radiographs 6-week: DICOM viewer	CT	Scaphoid	79 (72-85)	55 (51-58)	58 (55-61)	23 (19-27)	94 (91-96)
Mallee (2016) [57]	Radiographs 6-week: JPEG	MRI + CT	Scaphoid	52 (45-59)	58 (55-60)	57 (55-59)	14 (12-17)	90 (88-92)
Mallee (2016) [57]	Radiographs 6-week: DICOM viewer	MRI + CT	Scaphoid	75 (67-83)	53 (50-56)	56 (52-59)	18 (14-21)	94 (92-96)
Ottenin 2012 [60]	Radiographs	Clinical follow-up	Scaphoid	67ɸ	93ɸ	88ɸ	68ɸ	92ɸ
Ottenin 2012 [60]	Radiographs	Clinical follow-up	Other carpal bones	40ɸ	94ɸ	88ɸ	44ɸ	93ɸ
Brink (2019) [68]	X-ray	1-year clinical follow-up	Scaphoid	25	97			
	X-ray	1-year clinical follow-up	Triquetral	18	100			
	X-ray	1-year clinical follow-up	Lunate	0	100			
	X-ray	1-year clinical follow-up	Trapezium	0	100			
	X-ray	1-year clinical follow-up	Trapezoid	0	100			
	X-ray	1-year clinical follow-up	Hamate	100	100			
	X-ray	1-year clinical follow-up	Capitate	100	100			
Neubauer (2018) [69]	Radiography	Clinical follow-up	Scaphoid	87 (83-92)	77 (71-83)	82	80 (75-86)	84 (80-90)
Imaging: MRI								
Beeres (2008) [47]	MRI 1,5T (<24h)	A combination of MRI, bone scintigraphy and when not in agreement, clinical follow-up	Scaphoid	80 (56-94)	100 (96-100)	96	100 (74-100)	95 (88-99)
Kumar (2005) [55]	MRI 1,5T (<24h)	MRI in those without fracture at MRI <24h or no clinical signs of fracture	Scaphoid	100^b^	100^b^	100^b^	100^b^	100^b^
Mallee (2011) [56]	MRI 1.0T	Radiographs	Scaphoid	67	89	85	57 54^c^	93 93^d^
Memarsadeghi (2006) [59]	MRI 1,0T	Radiographs obtained 6 weeks after trauma.	All scaphoid	100 (82-100)	100 (87-100)	100	100	100
Memarsadeghi (2006) [59]	MRI 1,0T	Radiographs obtained 6 weeks after trauma.	Cortical scaphoid fractures	38 (16-65)	100 (52-100)	55 (24-85)	100	27
Memarsadeghi (2006) [59]	MRI 1,0T	Radiographs obtained 6 weeks after trauma.	Other carpal fractures	85	100	84		
de Zwart (2016) [66]	MRI (<72h)	Final diagnosis after MRI, CT, BS and 6-weeks clinical signs	Scaphoid	67	100 (88-100)	94	67	97
Imaging: (Multi detector) computed tomography								
Adey (2007) [43]	CT (first round interpretation)	Radiographs 6 weeks after injury	Scaphoid	89 (84-92)	91 (86-94)	89 (89-92)	28 (23-32)	99 (97-99)
	CT (second round interpretation)	Radiographs 6 weeks after injury	Scaphoid	97 (93-99)	85 (77-89)	88 (82-91)		
Breederveld (2004) [49]	CT	Clinical follow-up	Scaphoid	100	100	100	100	100
Cruickshank (2007) [50]	CT (same or next day)	The diagnosis on Day 10 with clinical examination and X-rays, with MRI performed in patients with persistent tenderness but normal X-rays.	Scaphoid and other fractures (Triquetral, Trapezium, Capitate and Lunate)	94 (72-100)	100 (87-100)	98	100 (78-100)	97 (82-100)
Ilica (2011) [54]	MDCT	MRI 1,5T	Scaphoid	86	100	95	100	91
Jorgsholm (2013) [72]	CT	MRI 0.23T (within 3 days)	Scaphoid	95 (91-97)				
	CT	MRI 0.23T (within 3 days)	Capitate	75 (35-97)				
	CT	MRI 0.23T (within 3 days)	Hamata	100 (40-100)				
Mallee (2011) [56]	CT	Radiographs	Scaphoid	67	96	91	80 76^c^	93 94^d^
Mallee (2014) [58]	CT-scaphoid: reformations in planes defined by the long axis of the scaphoid	Radiographs	Scaphoid	67	96	91	80 76^c^	93 94^d^
	CT-wrist: reformations made in the anatomic planes of the wrist	Radiographs	Scaphoid	33	89	79	40 36^c^	86 87^d^
Memarsadeghi (2006) [59]	MDCT	Radiographs obtained 6 weeks after trauma.	All scaphoid	73 (48-89)	100 (87-100)	89 (78-100)	100	86
Memarsadeghi (2006) [59]	MDCT	Radiographs obtained 6 weeks after trauma.	Cortical scaphoid fractures	100 (75-100)	100 (52-100)	100	100	100
Ottenin (2012) [60]	MDCT	Clinical follow-up	Scaphoid	77ɸ	94ɸ	91ɸ	76ɸ	95ɸ
Ottenin (2012) [60]	MDCT	Clinical follow-up	Other carpal bones	60ɸ	95ɸ	91ɸ	56ɸ	96ɸ
Rhemrev (2007) [63]	MDCT (<24h)	Final diagnosis after CT, BS and, both radiographic (6 weeks after injury) and physical reevaluation.	Scaphoid	64	99	94	90	94
de Zwart (2016) [66]	CT(<72h)	Final diagnosis after MRI, CT, BS and 6-weeks clinical signs	Scaphoid	33	100 (88-100)	94	100	94
Brink (2019) [68]	CT	1-year clinical follow-up	Scaphoid	100	100			
	CT	1-year clinical follow-up	Triquetral	100	100			
	CT	1-year clinical follow-up	Lunate	100	100			
	CT	1-year clinical follow-up	Trapezium	100	100			
	CT	1-year clinical follow-up	Trapezoid	100	100			
	CT	1-year clinical follow-up	Hamate	100	100			
	CT	1-year clinical follow-up	Capitate	100	0			
Neubauer (2018) [69]	CBCT	Clinical follow-up	Scaphoid	93 (89-96)	96 (93-99)	94	96 (93-99)	92 (89-96)
Borel (2017) [70]	CBCT	MRI	Scaphoid cortical fracture	100 (75-100)	97 (83-100)		94 (68-100)	100 (87-100)
	CBCT	MRI	All scaphoid fractures	94 (68-100)	97 (83-100)		94 (68-100)	97 (82-100)
	CBCT	MRI	Wrist cortical fracture	100 (83-100)	95 (75-100)		96 (78-100)	100 (83-100)
	CBCT	MRI	All wrist fractures	89 (70-97)	95 (75-100)		96 (78-100)	88 (67-97)
Imaging: Bone scintigraphy								
Beeres (2007) [46]	Bone scintigraphy (3-7 days after injury)	Clinical outcome	Scaphoid	92	87	88^a^	69^a^	97
	Bone scintigraphy (3-7 days after injury)	Clinical outcome	Scaphoid and other carpal bones	96	59^a^	80^a^	75	93^a^
Beeres (2008) [47]	Bone scintigraphy (between 3 and 5 days)	A combination of MRI, bone scintigraphy and when not in agreement, clinical follow-up	Scaphoid	100 (83-100)	90 (81-96)	92	71 (52-87)	100 (95-100)
Breederveld (2004) [49]	Bone scintigraphy (three-fase)	Clinical follow-up	Scaphoid	78	90	86	78	90
Rhemrev (2010) [62]	Bone scintigraphy (3-5 days)	Final diagnosis after CT, BS and, both radiographic (6 weeks after injury) and physical reevaluation.	Scaphoid	93	91	91	62	99
de Zwart (2016) [66]	Bone Scintigraphy (between 3 and5 days)	l diagnosis after MRI, CT, BS and 6-weeks clinical signs	Scaphoid	100	97 (83-100)	97	75	100
Imaging: Ultrasonography								
Fusetti (2005) [51]	HSR-S global evaluation	CT (immediately after HSR-S performed)	Scaphoid	100	79	83	56	100
	HSR-S scaphoid cortical disruption	CT (immediately after HSR-S performed)	Scaphoid	100	95	96	83	100
	HSR-S radioarpal (RS) effusion	CT (immediately after HSR-S performed)	Scaphoid	100	42	54	31	100
	HSR-S scapho-trapezium-trapezoid (STT) effusion	CT (immediately after HSR-S performed)	Scaphoid	100	84	88	62	100
	HSR-S cortical disruption with RS and STT effusion (high index of suspicion)	CT (immediately after HSR-S performed)	Scaphoid	100	100	100	100	100
Herneth (2001) [53]	US	MRI	Scaphoid	78	100	87	100	75
Javadzadeh (2014) [74]	BUS	Radiographs	Carpal bones	42 (23-64)	87 (74-94)	74 (62-83)	57 (33-79)	78 (65-88)
Javadzadeh (2014) [74]	WBT ultrasonography	Radiographs	Carpal bones	47 (27-68)	87 (74-94)	75 (64-84)	60 (36-80)	80 (67-89)
Platon (2011) [61]	US	CT	Scaphoid	92	71	76	46	97
	US	CT	Scaphoid fracture with a high potential of complication	100	67	71	30	100
Yildirim (2013) [65]	BUS	MRI (<24h)	Scaphoid	100 (69-100)	34 (19-52)	49	30 (16-49)	100 (74-100)
Imaging: Tomosynthesis								
Ottenin (2012) [60]	Tomosynthesis	Clinical follow-up	Scaphoid	91ɸ	98ɸ	96ɸ	90ɸ	98ɸ
Ottenin (2012) [60]	Tomosynthesis	Clinical follow-up	Other carpal bones	80ɸ	98ɸ	96ɸ	83ɸ	98ɸ
Scaphoid, other carpal bones and/or metacarpal fractures								
Physical examination								
Nikken (2005) [73]	Anatomic snuffbox tenderness	Additional treatment need	Scaphoid and other carpal bones. Metacarpal bones II–IV	39	78	62	56	65
Imaging: Radiographs								
Balci (2015) [71]	Radiographs	MDCT	Metacarpal	67	99		82	98
Jorgsholm (2013) [72]	Radiographs	MRI 0.23T (within 3 days)	Metacarpal	30 (7-65)				
Nikken (2005) [73]	Radiographs	Additional treatment need	Scaphoid and other carpal bones. Metacarpal bones II–IV	72	92	84	87	82
Brink (2019) [68]	X-ray	1-year clinical follow-up	Metacarpal	67	100			
Imaging: MRI								
Nikken (2005) [73]	MRI	Additional treatment need	Scaphoid and other carpal bones. Metacarpal bones II–IV	67	76	73	63	79
Imaging: CT								
Brink (2019) [68]	CT	1-year clinical follow-up	Metacarpal	100	100			
Metacarpal bones and finger fractures								
Physical examination								
Tayal (2007) [77]	Physical examination: deformity	Radiographs and surgical findings	Metacarpal bones and phalanx	55 (44-66)	89 (83-96)	76	77 (68-87)	75 (65-85)
	Physical examination: swelling	Radiographs and surgical findings	Metacarpal bones and phalanx	94 (88-99)	13 (5-20)	45	41 (30-52)	75 (65-85)
	Physical examination: erythema	Radiographs and surgical findings	Metacarpal bones and phalanx	26 (16-36)	85 (77-93)	62	53 (42-54)	63 (53-74)
Imaging: Ultrasonography								
Tayal (2007) [77]	US	Radiographs and surgical findings	Metacarpal bones and phalanx	90 (74-97)	98 (95-100)	95	97 (93-100)	94 (89-99)
Javadzadeh (2014) [74]	BUS	Radiographs	Metacarpal bones	73 (43-90)	78 (45-94)	70 (48-85)	80 (49-94)	70 (40-89)
	BUS	Radiographs	Phalanx	83 (61-94)	90 (78-96)	88 (78-94)	79 (57-91)	93 (81-97)
	WBT ultrasonography	Radiographs	Metacarpal bones	82 (52-95)	89 (57-98)	70 (48-85)	90 (60-98)	80 (49-94)
	WBT ultrasonography	Radiographs	Phalanx	94 (74-99)	95 (84-99)	95 (86-98)	89 (87-100)	98 (87-100)
Kocaoglu (2016) [76]	US	Radiographs	Metacarpal bones	93 (79-98)	98 (90-100)	96	97 (85-100)	95 (85-98)
Imaging: CBCT								
Faccioli (2010) [75]	CBCT	MSCT	Articular involvement of the phalanx	100	100	100	100	100
	CBCT	MSCT	Phalangeal bone fragments	87	100	92	100	82

*BUS* Bedside Ultra Sonography, *CBCT* Cone Beam Computed tomography arthrography, *MDCT* Multidetector Computed tomography, *MRI* Magnetic resonance imaging, *T* Tesla, *US* Ultra Sonography, *HSR-S* High Spatial Resolution sonography, *VAS* Visual Analogue Scale, *Se* Sensitivity, *Sp* Specificity, *PPV* Positive predictive value, *NPV* Negative predictive value, *LR* Likelihood ratio

^a^One patient had a physical examination matching with another carpal fracture instead of a scaphoid fracture at both 2 and 6 weeks after injury

^b^Four patient did not receive MRI during follow-up (reference standard)

^c^Positive predictive value accounting for prevalence and incidence

^d^Negative predictive value accounting for prevalence and incidence

^c/d^The positive predictive value and negative predictive value were determined with use of the Bayes theorem, which requires an a priori estimate of the prevalence (pretest probability) of the presence of scaphoid fractures. The positive predictive value is the patient’s probability of having a scaphoid fracture when the test is positive, and the negative predictive value is the probability of a patient not having a scaphoid fracture when the test is negative. The predictive values of any imaging modality depend critically on the prevalence of the characteristic in the patients being tested; hence the use of the appropriate Bayesian analysis is important. For the determination of positive and negative predictive values, we estimated an average prevalence of scaphoid fractures of 16% on the basis of the best available data. The positive predictive value was calculated as sensitivity · prevalence/(sensitivity · prevalence) 1 [(1 – specificity) · (1 – prevalence)], and the negative predictive value was calculated as specificity · (1 – prevalence)/[(1 – sensitivity) · prevalence] 1 [specificity · (1 – prevalence)].^54,60^

ɸ Average between presented individual values of three readers (junior radiologist, junior orthopedic surgeon and senior radiologist)

Repeated physical examination with radiological examination after 38 days [52] for diagnosing other carpal bone fractures showed a Se of 100% with the exception of the triquetrum (75%).

Radiographs used as an index test for diagnosing scaphoid fractures showed Se, Sp, accuracy, PPV and NPV ranging from 25 to 87%, 50–100%, 48–88%, 14–100% and 49–94%, respectively. For diagnosing scaphoid fractures, Magnetic Resonance Imaging (MRI) as an imaging modality showed Se, Sp, accuracy, PPV and NPV ranging from 67 to 100%, 89–100%, 85–100%, 54–100% and 93–100%, respectively. Multi Detector Computed Tomography (MDCT) showed Se, Sp, accuracy, PPV and NPV ranging from 33 to 100%, 85–100%, 79–100%, 28–100% and 86–100%, respectively. Bone Scintigraphy (BS) as an index test for diagnosing scaphoid fractures showed Se, Sp, accuracy, PPV and NPV ranging from 78 to 100%, 87–97%, 86–97%, 62–78% and 90–100%, respectively. For diagnosing scaphoid fractures, Ultrasonography (US) as an imaging modality showed Se, Sp, accuracy, PPV and NPV ranging from 78 to 100%, 34–100%, 49–100%, 30–100% and 75–100%, respectively.

### Diagnosing phalangeal and metacarpal fractures in hospital care

Table 5 also presents the accuracy of the diagnostic tests for metacarpal and/or phalangeal fractures, as described in six studies [71, 73–77]. Physical examination [77] for diagnosing phalangeal and metacarpal fractures showed Se, Sp, accuracy, PPV and NPV ranging from 26 to 55%, 13–89%, 45–76%, 41–77% and 63–75%, respectively. Imaging for metacarpal and finger fractures showed Se, Sp, accuracy, PPV and NPV ranging from 73 to 100%, 78–100%, 70–100%, 79–100% and 70–100%, respectively. The reported diagnostic accuracy measures of phalangeal and metacarpal fractures were characterized by markedly heterogeneous results among the eligible studies.

### Combined diagnostic accuracy of the studies with no limitations and no incorporation Bias

Table 6 shows combined diagnostic accuracy measures of the studies that had no limitations and no incorporation bias. A wide range of results were found for the specificity, accuracy and NPV of MRI, US, CT and BS. The sensitivity of BS and US showed similar, acceptable results. US and MRI are imaging tools that have similar PPV, but with large confidence intervals.

**Table 6:** Combined Diagnostic Accuracy of the Studies with no Limitations on QUADAS-2 and No Incorporation Bias (*N* = 7)

Author(s)	Diagnostic test	Scaphoid fracture	Se %	Sp %	Accuracy %	PPV %	NPV %
Gabler (2001) [52]	Repeated clinical and radiological examinations^a^	Scaphoid	82–100	100	100	100	100
Mallee (2016) [57]	Radiographs ^b^	Scaphoid	42–79	53–59	53–58	14–26	79–94
Fusetti (2005) [51] and Platon (2011) [61]	Ultrasonography	Scaphoid	92–100	42–100	54–100	30–100	97–100
Mallee (2011) [56]	MRI	Scaphoid	67	8	85	57	93
Mallee (2011) [56] and Mallee (2014 [58]	(MD)CT^c^	Scaphoid	33–67	89–96	79–91	40–80	86–93
Borel (2017) [70]	CBCT	Scaphoid	94	97		94	97
Author	Diagnostic test	Other carpal fracture	Sensitivity %	Specificity %	Accuracy %	PPV %	NPV %
Mallee (2014) [58]	Repeated clinical and radiological examinations	Other carpal bones	75–100				

^a^Repeated clinical and radiological examinations after 10 and 38 days

^b^Radiographs after 6 weeks evaluated with JPEG or DICOM files

^c^CT-scaphoid: reformations in planes defined by the long axis of the scaphoid versus CT-wrist: reformations made in the anatomic planes of the wrist

## Discussion

In previous reviews, no studies were identified on the diagnostic accuracy of history taking for phalangeal, metacarpal or carpal fractures. In the current systematic review, only two such studies were identified. This update included one extra study on physical examinations for diagnosing scaphoid fractures in hospital care, which was not included in previous reviews [48]. Based on these results and those presented in the previous reviews, physical examination is of moderate use for diagnosing a scaphoid fracture. Physicians should be aware that tenderness in the anatomical snuff box (ASB), tenderness over the scaphoid tubercle and pain on longitudinal compression of the thumb have limited added value in a diagnostic process for a scaphoid fracture.

The present systematic review identified eight supplementary imaging studies [58, 61, 65, 66, 68–70, 74], subdivided into MRI [66], CT [58, 66, 68–70], BS [66] and US [61, 65, 74]. The overall conclusion is that imaging tests were found to be moderately accurate for a definitive diagnosis. However, the standard diagnostic work-up for wrist complaints suspected of being a fracture should also include detailed patient history taking, a conscientious physical examination and, only if needed, imaging [23]. Diagnostic studies focusing on history taking and physical examination of patients with suspected phalangeal, metacarpal and carpal fractures are therefore desired.

Compared with previous reviews, the current systematic review attempted to distinguish between studies based on their setting. Remarkably, no studies examined the diagnostic accuracy of any diagnostic test for phalangeal, metacarpal and carpal fractures in a non-institutionalized general practitioner care setting. It is known that results from hospital care cannot automatically be translated into guidelines for non-institutionalized general practitioner care. For that reason, it is not possible to advise general practitioners properly on the diagnosis of carpal, metacarpal and phalangeal fractures based on the currently available literature. Given the burden of finger, hand and wrist fractures on non-institutionalized care and the importance of proper diagnoses, diagnostic studies focusing on phalangeal, metacarpal and carpal fractures in non-institutionalized general practitioner care are urgently needed [2].

### Methodological quality assessment

The methodological quality of the eligible studies included in this update was limited, which might affect the estimates of diagnostic accuracy. Many of the included studies had methodological flaws and lacked the necessary details to replicate the studies. There was considerable underreporting of important domains in most of the included studies. The studies in this and previous systematic reviews also had the inherent risk of publication bias. As the mechanisms of publication bias are not yet well understood for diagnostic accuracy studies, there are currently no assessment tools available to investigate this risk other than graphical interpretation. Furthermore, several studies demonstrate incorporation bias, with the risk of overestimation of the diagnostic accuracy [78].

### Diagnostic accuracy of the diagnostic tests for phalangeal and metacarpal fractures

The identified studies evaluated a variety of metacarpal and phalangeal pathologies. US may be an option for detecting metacarpal fractures and prevent unnecessary X-ray imaging examinations in patients presenting to the Emergency Department (ED) with hand trauma. Some advantages of US have increased its utilization in emergency departments; these include a short procedure time, a non-invasive and nonionizing radiation involving nature, availability for use in nonhospital settings or bedside settings, repeatability, and a higher safety in children and pregnant patients [79].

None of the previous reviews included studies showing evidence on the diagnostic accuracy for diagnosing metacarpal and phalangeal fractures. Therefore, this is the first study to systematically summarize the diagnostic accuracy of diagnostic tests for phalangeal and metacarpal fractures. This study concludes that physical examination was of limited use for diagnosing phalangeal and metacarpal fractures.

### Diagnostic accuracy of history taking and physical examination of carpal fractures

History taking and physical examination are important tools in a diagnostic process of diagnosing patients with wrist pain [23]. Although common practice in hospital care, only two studies were found on the diagnostic accuracy of history taking for carpal fractures in the previous reviews and current review.

Previous reviews reported that tenderness in the anatomical snuff box demonstrated an Se and Sp for scaphoid fractures ranging from 87 to 100% and 3–98%, respectively [32, 34]. Tenderness over the scaphoid tubercle (ST) demonstrated a Se and Sp ranging from 82 to 100% and 17–57%, respectively [32, 34]. The Longitudinal Thumb Compression test (LTC) demonstrated a Se and Sp ranging from 48 to 100% and 22–97%, respectively [32, 34].

The current systematic update included three extra studies on physical examinations for diagnosing scaphoid fractures in hospital care [48, 52, 53]. Based on these results and those presented in the previous reviews, combining provocative tests improved the accuracy of the post-test fracture probability, and physical examination alone was not sufficient to rule in or rule out scaphoid fracture, which may lead to unnecessary out-patient reviews and/or overtreatment. If a patient with wrist pain and normal X-rays has a combination of tenderness in the anatomical snuff box, tenderness over the scaphoid tubercle and longitudinal compression (LC) tenderness towards the scaphoid, supplementary imaging is still recommended. At present, in a patient with a strong suspicion of a scaphoid fracture based on history taking and physical examination despite no deviation on imaging, the wrist will be temporarily immobilized until repeated evaluation of the physical examination and imaging has taken place later [80].

### Diagnostic accuracy of imaging of carpal fractures

In this and previous systematic reviews, the reported diagnostic accuracy measures for imaging modalities were characterized by markedly heterogeneous results among the eligible studies. Plain radiography remained the commonest modality for diagnosing carpal fractures [81–83]. Its advantages include its wide availability, easy accessibility and low costs. Most studies describe diagnostic tests of scaphoid fractures and only a few studies concern other carpal fractures. At present, there is still insufficient scientific evidence regarding the ideal imaging technique for scaphoid fractures [23]. Repeated radiographs seems to have limited value for evaluating suspected scaphoid fractures. The irregular contour, the three-dimensional location in the wrist of the scaphoid and the overlap of the carpal bones render interpretation of scaphoid radiographs difficult, especially in the absence of fracture dislocation [81–83].

The best diagnostic modality for confirmation of the diagnosis of a carpal fracture that is not visible on the initial radiograph is still the subject of debate. As found in previous reviews (Table 1), MRI, CT and BS have been shown to have better diagnostic performance than isolated repeated scaphoid radiographs. Previous reviews by Yin et al. concluded that BS and MRI have equally high pooled sensitivity and high diagnostic value for excluding scaphoid fracture, when the lack of a reference standard is acknowledged [35, 36]. However, MRI is more specific and better for confirming scaphoid fractures when compared to BS. According to the Cochrane review of Mallee et al., statistically BS is the best diagnostic modality for establishing a definitive diagnosis in clinically suspected fractures when radiographs appear normal, but the number of overtreated patients is substantially lower with CT and MRI [39]. Moreover, physicians must keep in mind that BS is more invasive than the other modalities. Previous reviews by Kwee et al. and Ali et al. concluded that US can diagnose occult scaphoid fracture with a fairly high degree of accuracy and Kwee et al. stated that US may be used when CT and MRI are not readily available [37, 38]. Nonetheless, one needs to keep in mind that, although scaphoid fractures are the most frequently injured carpal bones, the consequences of fractures of other carpal bones should not be underestimated. All previously available systematic reviews only examined diagnostic tests for scaphoid fractures [32–39], while in practice it is often not quite clear during the diagnostic process which hand or wrist anatomical structure or tissue (soft tissue or bone) is affected.

## Conclusion

As no studies in non-institutionalized general practitioner care were identified, general practitioners who examine patients with a suspected hand or wrist fracture have limited instruments for providing adequate diagnostics. A general practitioner could decide to refer such patients to a hospital for specialized care, but one could question what assessments a specialist can use to come to an accurate diagnosis. In hospital care, two studies of the diagnostic accuracy of history taking for phalangeal, metacarpal and carpal fractures were found and physical examination was of moderate use for diagnosing a scaphoid fracture and of limited use for diagnosing phalangeal, metacarpal and remaining carpal fractures. Based on the best evidence synthesis, imaging tests (conventional radiograph, MRI, CT and BS) were only found to be moderately accurate for definitive diagnosis in hospital care.

## Data Availability

The datasets used and/or analysed during the current study are available from the corresponding author on reasonable request.

## Abbreviations

ASB: Anatomic snuff-box
BS: Bone scintigraphy
BUS: Bedside ultra sonography
CBCT: Cone beam computer tomography
CT: Computed tomography
HR: High risk
HSR-S: High spatial resolution-sonography
LR: Likelihood ratio
LTC: Longitudinal (thumb) compression test
MDCT: Multi detector computed tomography
MRI: Magnetic resonance imaging
MSCT: Multi-slice computer tomography
NPV: Negative predictive value
PPV: Positive predictive value
QUADAS: Quality Assessment of diagnostic accuracy studies
ROM: Range of motion
Se: Sensitivity
Sp: Specificity
STT: Scaphoid tubercle tenderness
T: Tesla
UR: Unclear Risk
US: Ultra sonography
VAS: Visual analogue scale
WBT: Water bath technique

## References

[1] Owen RA, Melton LJ, Johnson KA, Ilstrup DM, Riggs BL (1982). Incidence of Colles fracture in a north American community. Am J Public Health.

[2] Larsen CF, Mulder S, Johansen AM, Stam C (2004). The epidemiology of hand injuries in The Netherlands and Denmark. Eur J Epidemiol.

[3] McCullough NP, Smith FW, Cooper JG (2011). Early MRI in the management of the clinical scaphoid fracture. Eur J Emerg Med.

[4] van der Molen AB, Groothoff JW, Visser GJ, Robinson PH, Eisma WH (1999). Time off work due to scaphoid fractures and other carpal injuries in the Netherlands in the period 1990 to 1993. J Hand Surg Br.

[5] Hey HWD, Chong AKS, Murphy D (2011). Prevalence of carpal fracture in Singapore. J Hand Surg Am..

[6] Van Onselen EB, Karim RB, Hage JJ, Ritt MJ (2003). Prevalence and distribution of hand fractures. J Hand Surg Br.

[7] Cooney WP (2003). Scaphoid fractures: current treatments and techniques. Instr Course Lect.

[8] Krasin E, Goldwirth M, Gold A, Goodwin DR (2001). Review of the current methods in the diagnosis and treatment of scaphoid fractures. Postgrad Med J.

[9] Frazier WH, Miller M, Fox RS, Brand D, Finseth F (1978). Hand injuries: incidence and epidemiology in an emergency service. JACEP.

[10] Aitken S, Court-Brown CM (2008). The epidemiology of sports-related fractures of the hand. Injury.

[11] Van der Linden MW, Westert GP, de Bakker DH, Schellevis FG (2004). Tweede Nationale Studie naar ziekten en verrichtingen in de huisartspraktijk.

[12] Roolker W, Maas M, Broekhuizen AH (1999). Diagnosis and treatment of scaphoid fractures, can non union be prevented?. Arch Orthop Trauma Surg.

[13] Schaub TA, Chung KC (2006). Systems of provision and delivery of hand care, and its impact on the community. Injury..

[14] de Putter CE, van Beeck EF, Polinder S, Panneman MJ, Burdorf A, Hovius SE, Selles RW (2016). Healthcare costs and productivity costs of hand and wrist injuries by external cause: a population-based study in working-age adults in the period 2008-2012. Injury..

[15] Greene WB. Essentials of musculoskeletal care. Rosemont, IL: American Academy of Orthopaedic Surgeons, 2001.

[16] Langhoff O, Andersen JL (1988). Consequences of late immobilization of scaphoid fractures. J Hand Surg Br.

[17] Eddeland A, Eiken O, Hellgren E, Ohlsson NM (1975). Fractures of the scaphoid. Scand J Plast Reconstr Surg.

[18] Taleisnik J (1988). Clinical and technologic evaluation of ulnar wrist pain. J Hand Surg [Am].

[19] Steenvoorde P, Jacobi C, van der Lecq A, van Doorn L, Kievit J, Oskam J (2006). Development of a clinical decision tool for suspected scaphoid fractures. Acta Orthop Belg.

[20] Phillips TG, Reibach AM, Slomiany WP (2004). Diagnosis and management of scaphoid fractures. Am Fam Physician.

[21] Freeland P (1989). Scaphoid tubercle tenderness: a better indicator of scaphoid fractures?. Arch Emerg Med.

[22] Grover R (1996). Clinical assessment of scaphoid injuries and the detection of fractures. J Hand Surg Br.

[23] Groves AM, Kayani I, Syed R, Hutton BF, Bearcroft PP, Dixon AK, Ell PJ (2006). An international survey of hospital practice in the imaging of acute scaphoid trauma. AJR Am J Roentgenol.

[24] Hobby JL, Tom BD, Bearcroft PW, Dixon AK (2001). Magnetic resonance imaging of the wrist: diagnostic performance statistics. Clin Radiol.

[25] Tiel-van Buul MM, van Beek EJ, Borm JJ, Gubler FM, Broekhuizen AH, van Royen EA (1993). The value of radiographs and bone scintigraphy in suspected scaphoid fracture. A statistical analysis. J Hand Surg Br.

[26] Hunter JC, Escobedo EM, Wilson AJ, Hanel DP, Zink-Brody GC, Mann FA (1997). MR imaging of clinically suspected scaphoid fractures. AJR Am J Roentgenol.

[27] Furunes H, Vandvik PO (2009). Cast immobilisation for suspected scaphoid fractures. Tidsskr Nor Laegeforen.

[28] Cheung GC, Lever CJ, Morris AD (2006). X-ray diagnosis of acute scaphoid. J Hand Surg Br..

[29] Lozano-Calderon S, Blazar P, Zurakowski D, Lee SG, Ring D (2006). Diagnosis of scaphoid fracture displacement with radiography and computed tomography. J Bone Joint Surg Am.

[30] Steel N, Abdelhamid A, Stokes T, Edwards H, Fleetcroft R, Howe A, Qureshi N (2014). A review of clinical practice guidelines found that they were often based on evidence of uncertain relevance to primary care patients. J Clin Epidemiol.

[31] Ring D, Lozano-Calderon S (2008). Imaging for suspected scaphoid fracture. J Hand Surg Am.

[32] Carpenter CR, Pines JM, Schuur JD, Muir M, Calfee RP, Raja AS (2014). Adult scaphoid fracture. Acad Emerg Med.

[33] Burrows B, Moreira P, Murphy C, Sadi J, Walton DM (2014). Scaphoid fractures: a higher order analysis of clinical tests and application of clinical reasoning strategies. Man Ther.

[34] Mallee WH, Henny EP, van Dijk CN, Kamminga SP, van Enst WA, Kloen P (2014). Clinical diagnostic evaluation for scaphoid fractures: a systematic review and meta-analysis. J Hand Surg Am.

[35] Yin ZG, Zhang JB, Kan SL, Wang XG (2012). Diagnostic accuracy of imaging modalities for suspected scaphoid fractures: meta-analysis combined with latent class analysis. J Bone Joint Surg Br..

[36] Yin ZG, Zhang JB, Kan SL, Wang XG (2010). Diagnosing suspected scaphoid fractures: a systematic review and meta-analysis. Clin Orthop Relat Res.

[37] Kwee RM, Kwee TC (2018). Ultrasound for diagnosing radiographically occult scaphoid fracture. Skelet Radiol.

[38] Ali M, Ali M, Mohamed A, Mannan S, Fallahi F (2018). The role of ultrasonography in the diagnosis of occult scaphoid fractures. J Ultrason.

[39] Mallee WH, Wang J, Poolman RW, Kloen P, Maas M, de Vet HCW, Doornberg JN. Computed tomography versus magnetic resonance imaging versus bone scintigraphy for clinically suspected scaphoid fractures in patients with negative plain radiographs. Cochrane Database of Systematic Reviews 2015, Issue 6. Art. No.: CD010023. 10.1002/14651858.CD010023.pub2 PMC6464799 26045406

[40] Moher D, Liberati A, Tetzlaff J, Altman DG; PRISMA group Preferred reporting items for systematic reviews and meta-analyses: the PRISMA statement BMJ 2009; 339: b2535. PMC3090117 21603045

[41] Whiting PF, Rutjes AW, Westwood ME, Mallett S, Deeks JJ, Reitsma JB, Leeflang MM, Sterne JA, Bossuyt PM (2011). QUADAS-2: a revised tool for the quality assessment of diagnostic accuracy studies. QUADAS-2 group. Ann Intern Med.

[42] Devillé WL, Buntinx F, Bouter LM, Montori VM, de Vet HC, van der Windt DA, Bezemer PD (2002). Conducting systematic reviews of diagnostic studies: didactic guidelines. BMC Med Res Methodol.

[43] Adey L, Souer JS, Lozano-Calderon S, Palmer W, Lee SG, Ring D (2007). Computed tomography of suspected scaphoid fractures. J Hand Surg Am..

[44] Annamalai G, Raby N (2003). Scaphoid and pronator fat stripes are unreliable soft tissue signs in the detection of radiographically occult fractures. Clin Radiol.

[45] Behzadi C, Karul M, Henes FO, Laqmani A, Catala-Lehnen P, Lehmann W, Nagel HD, Adam G, Regier M (2015). Comparison of conventional radiography and MDCT in suspected scaphoid fractures. World J Radiol.

[46] Beeres FJ, Hogervorst M, Rhemrev SJ, den Hollander P, Jukema GN (2007). A prospective comparison for suspected scaphoid fractures: bone scintigraphy versus clinical outcome. Injury..

[47] Beeres FJ, Rhemrev SJ, den Hollander P, Kingma LM, Meylaerts SA, le Cessie S, Bartlema KA, Hamming JF, Hogervorst M (2008). Early magnetic resonance imaging compared with bone scintigraphy in suspected scaphoid fractures. J Bone Joint Surg Br.

[48] Bergh TH, Lindau T, Soldal LA, Bernardshaw SV, Behzadi M, Steen K, Brudvik C (2014). Clinical scaphoid score (CSS) to identify scaphoid fracture with MRI in patients with normal x-ray after a wrist trauma. Emerg Med J.

[49] Breederveld RS, Tuinebreijer WE (2004). Investigation of computed tomographic scan concurrent criterion validity in doubtful scaphoid fracture of the wrist. J Trauma.

[50] Cruickshank Jaycen, Meakin Alex, Breadmore Ross, Mitchell David, Pincus Steven, Hughes Thomas, Bently Bronwyn, Harris Mark, Vo Austin (2007). Early computerized tomography accurately determines the presence or absence of scaphoid and other fractures. Emergency Medicine Australasia.

[51] Fusetti C, Poletti PA, Pradel PH, Garavaglia G, Platon A, Della Santa DR, Bianchi S (2005). Diagnosis of occult scaphoid fracture with high-spatial-resolution sonography: a prospective blind study. J Trauma.

[52] Gäbler C, Kukla C, Breitenseher MJ, Trattnig S, Vécsei V (2001). Diagnosis of occult scaphoid fractures and other wrist injuries. Are repeated clinical examinations and plain radiographs still state of the art?. Langenbeck's Arch Surg.

[53] Herneth AM, Siegmeth A, Bader TR, Ba-Ssalamah A, Lechner G, Metz VM, Grabenwoeger F (2001). Scaphoid fractures: evaluation with high-spatial-resolution US initial results. Radiol.

[54] Ilica AT, Ozyurek S, Kose O, Durusu M (2011). Diagnostic accuracy of multidetector computed tomography for patients with suspected scaphoid fractures and negative radiographic examinations. Jpn J Radiol.

[55] Kumar S, O'Connor A, Despois M, Galloway H (2005). Use of early magnetic resonance imaging in the diagnosis of occult scaphoid fractures: the CAST Study (Canberra Area Scaphoid Trial). N Z Med J.

[56] Mallee W, Doornberg JN, Ring D, van Dijk CN, Maas M, Goslings JC (2011). Comparison of CT and MRI for diagnosis of suspected scaphoid fractures. J Bone Joint Surg Am.

[57] Mallee WH, Mellema JJ, Guitton TG, Goslings JC, Ring D (2016). Doornberg JN; science of variation group. 6-week radiographs unsuitable for diagnosis of suspected scaphoid fractures. Arch Orthop Trauma Surg.

[58] Mallee WH, Doornberg JN, Ring D, Maas M, Muhl M, van Dijk CN, Goslings JC (2014). Computed tomography for suspected scaphoid fractures: comparison of reformations in the plane of the wrist versus the long axis of the scaphoid. Hand (NY).

[59] Memarsadeghi M, Breitenseher MJ, Schaefer-Prokop C, Weber M, Aldrian S, Gäbler C, Prokop M (2006). Occult scaphoid fractures: comparison of multidetector CT and MR imaging-initial experience. Radiol.

[60] Ottenin MA, Jacquot A, Grospretre O, Noël A, Lecocq S, Louis M, Blum A (2012). Evaluation of the diagnostic performance of tomosynthesis in fractures of the wrist. AJR Am J Roentgenol.

[61] Platon A, Poletti PA, Van Aaken J, Fusetti C, Della Santa D, Beaulieu JY, Becker CD (2011). Occult fractures of the scaphoid: the role of ultrasonography in the emergency department. Skelet Radiol.

[62] Rhemrev SJ, Beeres FJ, van Leerdam RH, Hogervorst M, Ring D (2010). Clinical prediction rule for suspected scaphoid fractures: A prospective cohort study. Injury..

[63] Rhemrev SJ, de Zwart AD, Kingma LM, Meylaerts SA, Arndt JW, Schipper IB, Beeres FJ (2010). Early computed tomography compared with bone scintigraphy in suspected scaphoid fractures. Clin Nucl Med.

[64] Steenvoorde P, Jacobi C, van Doorn L, Oskam J (2006). Pilot study evaluating a clinical decision tool on suspected scaphoid fractures. Acta Orthop Belg.

[65] Yıldırım A, Unlüer EE, Vandenberk N, Karagöz A (2013). The role of bedside ultrasonography for occult scaphoid fractures in the emergency department. Ulus Travma Acil Cerrahi Derg.

[66] de Zwart AD, Beeres FJ, Rhemrev SJ, Bartlema K, Schipper IB (2016). Comparison of MRI, CT and bone scintigraphy for suspected scaphoid fractures. Eur J Trauma Emerg Surg.

[67] Sharifi MD, Moghaddam HZ, Zakeri H, Ebrahimi M, Saeedian H, Hashemian AM (2015). The accuracy of pain measurement in diagnosis of scaphoid bone fractures in patients with magnetic resonance imaging: report of 175 cases. Med Arch.

[68] Brink M, Steenbakkers A, Holla M, de Rooy J, Cornelisse S, Edwards MJ, Prokop M (2019). Single-shot CT after wrist trauma: impact on detection accuracy and treatment of fractures. Skelet Radiol.

[69] Neubauer J, Benndorf M, Ehritt-Braun C, Reising K, Yilmaz T, Klein C, Zajonc H, Kotter E, Langer M, Goerke SM (2018). Comparison of the diagnostic accuracy of cone beam computed tomography and radiography for scaphoid fractures. Sci Rep.

[70] Borel C, Larbi A, Delclaux S, Lapegue F, Chiavassa-Gandois H, Sans N, Faruch-Bilfeld M (2017). Diagnostic value of cone beam computed tomography (CBCT) in occult scaphoid and wrist fractures. Eur J Radiol.

[71] Balci A, Basara I, Çekdemir EY, Tetik F, Aktaş G, Acarer A, Özaksoy D (2015). Wrist fractures: sensitivity of radiography, prevalence, and patterns in MDCT. Emerg Radiol.

[72] Jørgsholm P, Thomsen NO, Besjakov J, Abrahamsson SO, Björkman A (2013). The benefit of magnetic resonance imaging for patients with posttraumatic radial wrist tenderness. J Hand Surg Am.

[73] Nikken JJ, Oei EH, Ginai AZ, Krestin GP, Verhaar JA, van Vugt AB, Hunink MG (2005). Acute wrist trauma: value of a short dedicated extremity MR imaging examination in prediction of need for treatment. Radiol.

[74] Javadzadeh HR, Davoudi A, Davoudi F, Ghane MR, Khajepoor H, Goodarzi H, Faraji M, Mahmoudi S, Shariat SS, Emami MK (2014). Diagnostic value of "bedside ultrasonography" and the "water bath technique" in distal forearm, wrist, and hand bone fractures. Emerg Radiol.

[75] Faccioli N, Foti G, Barillari M, Atzei A, Mucelli RP (2010). Finger fractures imaging: accuracy of cone-beam computed tomography and multislice computed tomography. Skelet Radiol.

[76] Kocaoğlu S, Özhasenekler A, İçme F, Pamukçu Günaydın G, Şener A, Gökhan Ş (2016). The role of ultrasonography in the diagnosis of metacarpal fractures. Am J Emerg Med.

[77] Tayal VS, Antoniazzi J, Pariyadath M, Norton HJ (2007). Prospective use of ultrasound imaging to detect bony hand injuries in adults. J Ultrasound Med.

[78] Worster A, Carpenter C (2008). Incorporation bias in studies of diagnostic tests: how to avoid being biased about bias. CJEM..

[79] William DM, Kurtz BK, Hertzberg BS. In: Yımaz C, editor. Bilinmesi Gerekenler-Ultrason. Çev. Ed. 2. Baskı. İzmir: Güven Bilimsel; 2008:3–4.

[80] Dias J, Kantharuban S (2017). Treatment of scaphoid fractures: European approaches. Hand Clin.

[81] Low G, Raby N (2005). Can follow-up radiography for acute scaphoid fracture still be considered a valid investigation?. Clin Radiol.

[82] Munk B, Frokjaer J, Larsen CF, Johannsen HG, Rasmussen LL, Edal A, Rasmussen LD (1995). Diagnosis of scaphoid fractures. A prospective multicenter study of 1,052 patients with 160 fractures. Acta OrthopScand.

[83] Tiel-van Buul MM, van Beek EJ, Broekhuizen AH, Nooitgedacht EA, Davids PH, Bakker AJ (1992). Diagnosing scaphoid fractures: radiographs cannot be used as a gold standard!. Injury..

